# Cultivation of Lovage under Exposure of Light-Emitting Diode Illumination and Analysis of Rutin Produced by High Performance Liquid Chromatography (HPLC) and Ultraviolet-Visible Spectroscopy (UV-Vis)

**DOI:** 10.1155/2022/6357893

**Published:** 2022-11-17

**Authors:** Holger Wack, Annette Somborn, Dennis Schlehuber, Stephan Deckert, Volkmar Keuter

**Affiliations:** Fraunhofer Institute for Environmental, Safety, and Energy Technology UMSICHT, Osterfelder Strasse 3, 46047 Oberhausen, Germany

## Abstract

To meet the requirements of a sustainable agricultural economy such as quality, resource conservation, and efficiency, the interaction between the cultivation parameters of plants and the resulting plant ingredients are becoming more and more interesting, especially regarding secondary metabolites. Therefore, the cultivation of lovage under the influence of light-emitting diode illumination and controlled environment conditions was studied and data on the rutin concentrations obtained are presented. Different extraction agents and processes were tested for the treatment of the leaves and the rutin was analyzed using high performance liquid chromatography (HPLC) and ultraviolet-visible spectroscopy (UV-Vis spectroscopy). UV-Vis spectroscopy was found to provide a simple and rapid method of predicting the rutin concentration. The significant parameter regarding the rutin concentration obtained was light intensity and overall rutin concentrations in the range of 17,005 mg to 34,759 mg of rutin per kg dry leaves were found.

## 1. Introduction

The key conditions for successful plant production are the availability of light, water, heat, and nutrients. The requirements for quality, resource conservation, and efficiency are increasing, especially in agriculture, and are components of a sustainable agricultural economy. The knowledge of the plants' needs for the above-mentioned factors has mainly arisen from the many years of experience of specialized horticultural companies. Short-term, flexible, and foresighted adjustments to the quite changing needs of plants are not always possible.

The growth of plants is decisively influenced by light [1]. However, photosynthesis does not require the entire sunlight spectrum, only the range of blue light (approx. 420-480 nm) and red light (approx. 630-790 nm). New, powerful light-emitting diodes (LEDs) offer enormous potential in plant cultivation, as it is possible to emit only the required spectrum. Once the necessary light scenario under which the desired secondary compounds are best formed is known, the appropriate LED can be selected and the respective intensities adjusted. Depending on the required light scenarios, the appropriate LEDs can be selected and thus the optimal exposure spectrum for each plant can be compiled, especially regarding the content and concentration of secondary plant compounds.

Secondary plant compounds are chemical compounds formed by plants in their secondary metabolism [2] and are used by plants to defend themselves against pests or predators and as protective substances against environmental influences such as strong solar radiation [3]. Since plants, unlike animals, are sessile, they produce a particularly large number of these substances [4, 5]. It is estimated that there are more than 60,000 different compounds. The concentration of many substances in individual plant parts is sufficient to have a toxic effect on pests. For humans, on the other hand, many of these act as flavoring or fragrances.

Rutin is a flavonoid and a glycoside of quercetin with the disaccharide rutinose, which is composed of rhamnose and glucose. Rutin is produced by many plants, mainly in the leaves, as a pigment to protect against UV radiation. Many flavonoids have been shown to have an antioxidant effect in humans; rutin is a major ingredient of several nutraceuticals and targets the blood vessels and the intestine in particular [6, 7].

In this work, an extraction and analysis method is described for lovage with respect to the secondary plant constituent rutin. Rutin was chosen because of the medical relevance, the availability of standard substances for the development of calibration methods, and the option to be analyzed by high performance liquid chromatography (HPLC). Lovage is known to contain rutin and own pretests had shown that lovage can be cultivated successfully under controlled environment conditions in climate chambers. The rutin concentration was adjusted within a wide range by choosing different LED illumination exposure scenarios. High performance liquid chromatography (HPLC) and UV-Vis spectroscopy were used to analyze the rutin.

## 2. Materials and Methods

### 2.1. Cultivation of Lovage and LED Illumination

Lovage (*Levisticum officinale*) belongs to the umbellifer family. The plant forms perennials of up to 2 m in height and originally comes from the Iran/Afghanistan region. Both the dried roots and the pinnate leaves can be used for seasoning and medicinal purposes. Lovage grows well in semishaded areas and prefers moderately nutrient-rich, moderately moist, and slightly acidic to neutral soils.

For the current investigation, lovage seeds from the company Sperli GmbH (Everswinkel, Germany) were used. The plants were cultivated using long day conditions (day : night = 16 : 8 hours) by illumination with fluorescent tubes at an intensity of 90 *μ*mol m^−2^ s^−1^. The cultivation was carried out under controlled environment conditions in a climate chamber (Climate Chambers: CLF Plant Climatics®, models E36 and E41, Wertlingen, Germany). For the cultivation, a relative humidity of 70% was set and a temperature of 18°C was used at night and 23°C by day. Watering was carried out daily and no fertilizer was applied. After the growing phase, the plants were thinned out, which means that only the strongest plant remained in the pot. The removed plants were extracted and analyzed. The ingredient concentrations obtained were used as a starting value for the exposure experiments. Subsequently, the trays with the remaining plants were placed under the light exposure.

The LED modules were from the company Heliosspectra, model LX206C, Gothenburg, Sweden. The light intensity (PPFD, photosynthetically photon flux density) was varied using two target intensities of 75 and 275 *μ*mol m^−2^ s^−1^. Based on the control and regulation system of the LED modules, the target intensities could be reached with a variation of ±25 *μ*mol m^−2^ s^−1^. In addition to the variation of the PPFD, the green, red-blue, and infrared components were varied. As reported by Thoma et al. [2] blue wavelengths mainly address the cryptochromes. The phytochromes are addressed in the green, red, and infrared range. Based on this, the following light variations were chosen: The green proportion was set to 20% and 40%, the red-blue ratio was varied at 1.0 : 0.5, 1.0 : 0.7, and 1.0 : 2.0, and the infrared proportion was set to 15% and 30%.

During the exposure phase, the plants were watered and fertilized from Monday to Friday. For this purpose, the plants were taken out of the chambers and 2,000 ml of a water-fertilizer solution with a conductivity of 1.5 mS was poured into the watering tray. The fertilizer solution was prepared using a NPK fertilizer (nitrate, phosphorus, and potassium, Hakaphos® Blue 15 + 10 + 15(+2), Compo Expert GmbH, Münster, Germany) using a conductivity meter.

### 2.2. Extraction

The leaves harvested were freeze-dried. They were then placed without disintegration in a vessel filled with the extraction agent. The ratio of leaves to extractant was 1 : 2 (w/w). Different mixtures of ethanol, methanol, and water (deionized water, Milli-Q®, Type I) were tested, each containing 1 wt. % of hydrochloric acid at a concentration of 37 wt. %. Afterwards, the mixture of leaves and extraction agent were immediately crushed with an Ultra-Turrax® (T50, Ika Werke GmbH, Staufen, Germany). By using this method, it was ensured that no substances were lost beforehand due to the effects of light, oxygen, or temperature. The extraction was carried out at 20°C for 2 hours and in the absence of oxygen. The resulting extraction agents were investigated with and without centrifugation. The aim of centrifugation was to obtain a maximum amount of extract and to prove that centrifugation will not affect the extraction process. This can be an important issue against the background of further process development and engineering. All of the substances used were of analytical grade (Sigma Aldrich Chemie GmbH, Germany).

### 2.3. High Performance Liquid Chromatography (HPLC)

**Figure 1:**
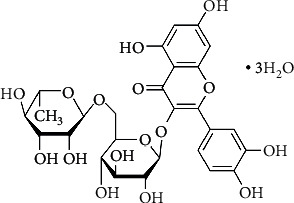
Rutin trihydrate used as standard.

The HPLC analytic was performed using an Agilent 1200 Series Gradient HPLC with a diode array detector (Agilent Technologies Inc., Santa Clara, USA) with an Agilent Poroshell 120 column EC-C18-C18 4.6, 150 mm, 4 *μ*m with a AJO-4286 SecurityGuard C18 4x2.0 mm (Phenomenex Ltd., Aschaffenburg, Germany). As an eluent, methanol/acetonitrile/water (40/15/45 wt. %) containing 1 wt. % acetic acid was used with a flow rate of 0.8 ml min^−1^. The oven temperature was 35°C and the diode array detector was set to 368 nm. As a standard, rutin trihydrate was used, Figure 1 (analytical grade, CAS-No. 153-18-4, Sigma Aldrich Chemie GmbH, Germany).

The concentrations used for calibration were 1, 5, 10, 20, 50, and 100 mg l^−1^. The retention time for rutin was 2.7 min. Injection volumes for rutin and the extraction solutions were 10 *μ*l and the extraction solutions were diluted 1 : 10 with water before analysis (deionized water, Milli-Q®, Type I).

### 2.4. Ultraviolet-Visible Spectroscopy (UV-Vis Spectroscopy)

UV-Vis scanning was carried out using a Shimadzu UV-2102 PC (Shimadzu Germany GmbH, Duisburg, Germany) with cuvettes of quartz glass for the sample and reference measurement. Analogous to the HPLC method, rutin trihydrate was used as a standard for calibration. The solution medium for this standard was the resulting extraction used for extraction (49.5 wt. % water, 49.5 wt. % ethanol, and 1 wt. % hydrochloric acid). Rutin concentrations of 1, 5, 10, 20, and 50 mg l^−1^ were prepared using the rutin trihydrate; the solution medium without rutin trihydrate was used as the reference.

## 3. Results and Discussion

### 3.1. Comparison of Extraction Methods

In the literature, there are many different approaches for extracting flavonoids from plants or plant parts [8–14]. These differ in terms of solvent, temperature, state of the plant (fresh, dried, frozen, and freeze-dried), and the extraction procedure itself.

This work aspired to avoid the use of solvents that are harmful to the environment and/or humans, such as dichloromethane or chloroform [15]. Thus, different extraction agents based on ethanol, methanol, and water as well as mixtures of these were tested, with addition of hydrochloric acid described by Złotek et al. [16] and Xie et al. [17] (Table 1). A batch of lovage obtained by a preliminary experiment of cultivation was used for the extraction experiments.

Compared to pure water, all extraction agents based on methanol and ethanol and centrifugation of the extraction solution resulted in more than double the yield. To keep the extraction method as simple as possible and for chemical toxicity reasons, the ethanol: water mixture was selected as the extraction agent and was also tested without centrifugation. The yield obtained was in the same range as with centrifugation. Therefore, an ethanol-water mixture (1 : 1, wt./wt.) with the addition of 1 wt. % hydrochloric acid (concentration 37 wt. %) was ultimately used and cold extraction (20°C) without centrifugation was performed for the further work. The temperature of 20°C was chosen to avoid a possible loss of metabolite during extraction through higher temperature [18].

### 3.2. Calibration of UV-VIS Scanning Spectroscopy

**Figure 2:**
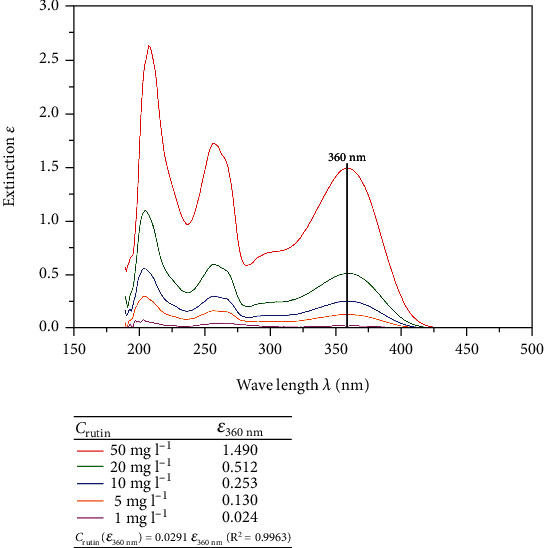
UV-Vis scanning for rutin by variation in the rutin concentration. To calibrate and subsequently measure the extraction samples, the absorbance maximum at a wavelength of 360 nm was chosen.

The full absorbance spectra of the rutin standards were determined by scanning in the wavelength range from 150 to 500 nm (Figure 2) and yielded stable absorbance lines with significant differences in absorbance as a function of rutin concentrations.

A linear fit of the values (fixed intercept at *c*_rutin_(0) = 0, least squares regression) yields the following linear function (determination coefficient *R*^2^ = 0.9963):

(1) $$\begin{array}{c} c_{\text{rutin}}\left(\varepsilon_{360nm}\right)=0.0291\;\varepsilon_{360nm},\text{where by }c_{\text{rutin}}\text{ is given in mg }{\text{l}}^{-1}. \end{array}$$

### 3.3. Comparison of Results Obtained via HPLC and UV-Vis

The extraction solvents obtained from the extraction of the plants that had been grown under different light exposures were analyzed using HPLC and UV-Vis.

**Figure 3:**
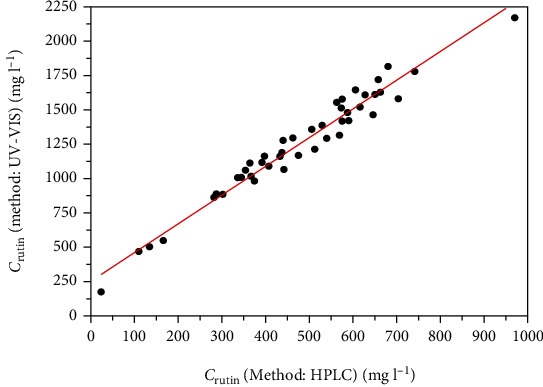
Concentration of rutin obtained by UV-Vis as a function of the rutin concentration obtained by HPLC for the extraction solvents.

The HPLC method delivers the specific rutin concentration for the different extraction solvents. Values from 168 mg l^−1^ to 973 mg l^−1^ were obtained (Figure 3). For the UV-Vis spectroscopy concentrations from 173 mg l^−1^ to 2,271 mg l^−1^ were obtained.

Using a linear fit, the correlation between the rutin concentration obtained by HPLC *c*_rutin/HPLC_ and the rutin concentration obtained by UV-Vis (HPLC) and *c*_rutin/UV−Vis_ can be expressed with the following function (determination coefficient *R*^2^ = 0.958):

(2) $$\begin{array}{c} c_{\text{rutin}/\text{HPLC}}=2.086\;c_{\text{rutin}/\text{UV}-\text{Vis}}+251.851. \end{array}$$

In contrast to the HPLC method, which specifically provides the rutin concentration quantitatively, UV-Vis spectroscopy determines a sum parameter of all plant compounds that absorb at 360 nm. This is evident from the higher concentration values that were obtained by the UV-Vis method compared to values obtained by the HPLC method.

However, taking into account that the UV-Vis method is much less complex than the HPLC method, the UV-Vis spectroscopy provides a simple and rapid method to predict the level of rutin in lovage, based on the correlation with the quantitatively HPLC results shown in Figure 3.

The interaction between the concentrations of the other plant compounds and the rutin concentration at different light exposures will be subject of further work, carried out by fractionation of the extraction solvents obtained and accompanying HPLC analysis.

### 3.4. Influence of LED Illumination on the Rutin Concentration

**Figure 4:**
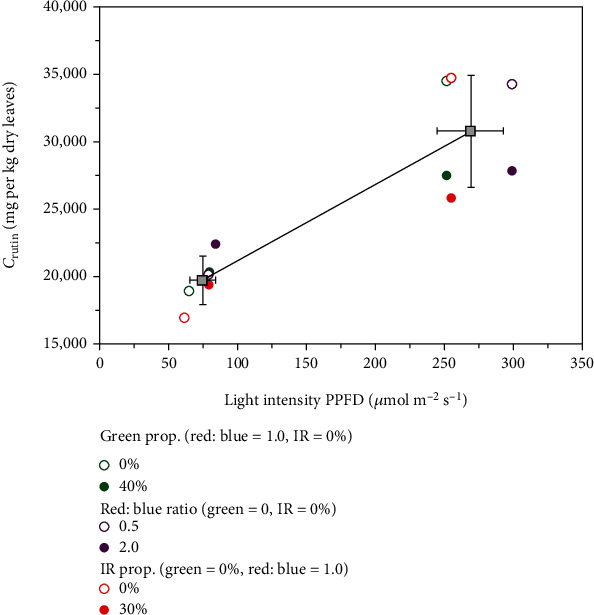
Concentration of rutin as a function of the light intensity by variation of light components. Dots with error ranges present the mean values of the rutin concentrations regarding the PPFD levels of 75 and 275 *μ*mol m^−2^ s^−1^.

In Figure 4 the rutin concentrations obtained are shown as a function of the PPFD value by variation of the green, red-blue, and infrared components. Standard deviations of the measurements were in the range of 15% regarding the rutin concentrations, for sake of clarity not shown. The standard deviations lead to an overlap of error ranges. Thus, in this work, no clear significances could be obtained for the influences of the green, red-blue, and infrared light components. Based on this results no further discussion is possible regarding the interactions of the light components and the amount of rutin formed in the plant.

Taking the mean value for all measurements at the PPFD level of 75 *μ*mol m^−2^ s^−1^ and 275 *μ*mol m^−2^ s^−1^, which is presented by the dots shown with error bars, leads to a significant influence of PPFD on the rutin concentration. Between the lowest PPFD level (75 *μ*mol m^−2^ s^−1^) and the highest PFFD level (275 *μ*mol m^−2^ s^−1^), the mean rutin concentration increases from 19,715 mg per kg of dry leaves to 30,759 mg per kg dry leaves. Overall, the lowest value found was 17,005 mg per kg of dry leaves and the highest value was 24,759 mg per kg of dry leaves.

In recent years, numerous plants have been investigated with regard to the rutin concentrations they contain. An overview of different studies is shown in Table 2. For better comparability, the concentrations were converted from the original data into the unit mg rutin per kg dry weight. The objectives of the studies were the variation of plant species, parts of plant, elicitation during cultivation with jasmonic acid and yeast extract, times of harvesting, use of different extraction agents, and the general screening for the presence of rutin. High concentrations of rutin, up to 121,750 mg per kg dry weight of inflorescences together with leaves, were found in buckwheat. Concentrations higher than 30,000 mg per kg dry weight are reported for amaranth, pichi pichi, and St. John's wort. For lovage concentrations in the range of 9,850 to 18,600 mg per kg dry weight (leaves) were found. All other plants studied contained concentrations less than 1,000 mg per kg dry weight. The reported concentrations of lovage are in the lower range of concentrations found in this work. In comparison, the highest concentrations found in this work are about a factor of 2 higher.

Against the background of plant production optimized for rutin, lovage can be deemed to be good-yielding. Based on the results obtained in this work, the plant can, to a large extent, be controlled in terms of the resulting rutin concentration by the light intensity used. In this work, an increase of the rutin concentration formed in the plant was found by increasing light intensity.

With regard to the energy consumption of the LED illumination, it is interesting to relate the total amount of rutin obtained to the amount of energy used. For this purpose, in addition to the rutin concentration *c*_rutin_, the corresponding mass of leaves *m*_leaves_ harvested must also be taken into account. Thus, PPFD specific rutin amounts *m*_Rutin/PPFD_ can be obtained as follows:

(3) $$\begin{array}{c} m_{\text{rutin}/\text{PPFD}}=c_{\text{rutin}}m_{\text{leaves}}{\text{PPFD}}^{-1}\left[\text{mg }{\left(\mu {\text{molm}}^{-2}{\text{s}}^{-1}\right)}^{-1}\right]. \end{array}$$

In Table 3 the results for the PPFD specific rutin amounts for the two levels of light intensity are shown.

It can be seen that the mass of leaves harvested and the concentrations of rutin obtained increase by increasing the light intensity. But looking at the PPFD specific rutin concentrations it becomes apparent that in terms of LED energy consumption the PPFD level of 275 *μ*mol m^−2^ s^−1^ is less efficient in comparison to the PPFD level of 75 *μ*mol m^−2^ s^−1^. It can be assumed that maybe some part of the energy at PPFD level of 275 *μ*mol m^−2^ s^−1^ is used for further metabolic reactions, e.g. photosynthesis and biomass build-up, or that the rutin quantities produced may be saturated. This opens up optimization potential for further investigations with regard to the amount of rutin obtained in interaction with the light intensity used.

## 4. Conclusion

This work presents the cultivation of lovage under the influence of LED illumination and the study of two analytical methods, high performance liquid chromatography (HPLC) and ultraviolet-visible spectroscopy (UV-Vis), for the rutin produced by the plant.

The cultivation was carried out under controlled environmental conditions at 70% relative humidity, a day: night ratio of 16 : 8 hours and temperatures of 23°C (day) and 18°C (night). To stimulate different concentrations of rutin in the lovage leaves, variations in light intensity, the green proportion, the red: blue ratio, and the infrared proportion were applied. From the results obtained, it can be concluded that the combination of different light components is far less important than the light intensity.

To analyze the rutin concentration, an extraction procedure was established using an ethanol: water mixture (1 : 1 wt./wt.) as the extraction agent with the addition of 1 wt. % hydrochloric acid. The extraction was carried out as a cold extraction under exclusion of oxygen at 20°C for 2 hours.

The obtained extraction solutions were analyzed using high performance liquid chromatography (HPLC) and ultraviolet-visible spectroscopy (UV-Vis spectroscopy) with respect to the rutin concentration. A linear relationship was found between the results of the two analytical methods. UV-Vis spectroscopy yields higher values than the HPLC method, which provides the specific rutin concentration. In addition to rutin, UV-Vis spectroscopy thus detects other plant constituents that are present at the same absorption maximum (360 nm) as rutin, but UV-Vis spectroscopy is suitable as a simple and rapid method for qualitatively predicting the rutin concentration.

Light intensity as photosynthetically photon flux density (PPFD) was identified as a significant parameter regarding the generation of rutin in the plant. Light intensity was varied at two levels (75 and 275 *μ*mol m^−2^ s^−1^), providing an overall concentration range of 17,005 mg of rutin per kg of dry leaves to 34,759 mg of rutin per kg of dry leaves. Lovage was identified as a good-yielding plant with respect to the secondary metabolite, rutin with the content depending significantly on the intensity of light exposure. But by calculating PPFD specific rutin amounts it was found that the rutin amount increases under proportional regarding the increase of light intensity.

In addition to that, the relationship between the results obtained by HPLC and UV-Vis spectroscopy have to be studied in more detail in further work, with particular emphasis on identifying the other constituents present in the plant in addition to rutin. The results are valuable not only for the indoor cultivation of plants in general, but also for analytics. When HPLC becomes replaceable by UV-Vis, sum parameters of plant constituents can be determined quickly and cheaply. This can be helpful for investigations with broad variations of plant types and cultivation parameters.

Finally, we note that the analysis of plants' secondary metabolites can provide a substantial amount of cultivation parameters like temperature, humidity, content of nutrients, and irrigation that can open up automation alternatives in cultivation unknown today. Using relatively simple and rapid analysis or even newly developed sensor technologies can aid in decision-making and hence lead to increased yields.

## Figures and Tables

**Table 1:** Overview of extraction agents, extraction process, and rutin concentrations obtained.

Extraction agent	Composition [wt.]	Centrifugation	Rutin concentration [mg per kg dry leaves]
Water	1	Yes	3,870
Ethanol: water	1 : 1	Yes	9,680
Methanol: water	1 : 1	Yes	9,650
Methanol: ethanol	1 : 1	Yes	9,720
Ethanol: water	1 : 1	No	10,100

**Table 2:** Values of rutin concentrations found in different plants. DW: dry weight. Part of plant investigated: f: flowers, i: inflorescences, l: leaves, s: seeds, st: stems.

Plant	Part of plant	Rutin concentration		Source
		[mg per kg DW]		
		Min	Max	
Amaranth (*amaranthus)*	f + l + s + st	2,600	38,000	[19]
Basil (*occimum basilicum)*	l	—	28	[20]
Buckwheat (*fagopyrum esculentum)*	l	11,000	112,000	[21]
Buckwheat (*fagopyrum esculentum)*	i + l	55,000	121,750	[22]
Buckwheat (*fagopyrum tataricum)*	l	—	3,190	[23]
Buckwheat (*fagopyrum tataricum)*	f	—	7,100	[23]
Calendula (*calendula officinalis)*	Fl	—	281	[20]
Celery (*apium graveolens)*	l	—	783	[24]
Dandelion (*taraxacum officinale)*	l	—	9,149	[20]
Chamomille (*matricaria chamomilla)*	f	—	311	[20]
Dill (*anethum graveolens)*	l + st	—	1,799	[24]
Fennel (*foeniculum vulgare)*	s	—	130	[20]
Green tea (*camellia sinensis)*	l	—	1,335	[20]
Lovage (*levisticum officinale)*	l	9,850	18,600	[16]
Lovage (*levisticum officinale)*	l	—	40	[25]
Lovage (*levisticum officinale)*	l	—	4,049	[26]
Lovage (*levisticum officinale)*	l	—	5,798	[24]
Parsley (*petroselinum crispum)*	l	—	297	[24]
Pichi pichi (*fabriana imbricata)*	l	9,900	33,500	[27]
St. John's wort (*hypericum perforatum)*	f	1,700	36,400	[28]

**Table 3:** Results for the PPFD specific rutin amounts based on the mean values of the levels of light intensity, the mass of leaves harvested (DW: dry weight), and the concentrations of rutin obtained.

PPFD level [*μ*mol m^−2^ s^−1^]	*m*_*l*eaves_ [mg DW]	*c*_rutin_ [mg per kg DW]	*m*_rutin/_ _PPFD_ [mg (*μ*mol m^−2^ s^−1^)^−1^]
75	211.8	22,643	6.4
275	262.0	32,110	3.6

## Data Availability

The data used to support the findings of this study are available from the corresponding author upon request.
